# Dynamics and interactions of cobalamin and folate status during advanced aging – a longitudinal study in a community-dwelling cohort with multiple follow-ups

**DOI:** 10.1186/s12937-020-00576-2

**Published:** 2020-07-02

**Authors:** Alexandra Jungert, Carola Zenke-Philippi, Monika Neuhäuser-Berthold

**Affiliations:** 1 Institute of Nutritional Science, Justus Liebig University, Goethestrasse 55, D-35390 Giessen, Germany; 2 Present address: Interdisciplinary Research Center for Biosystems, Land Use and Nutrition (IFZ), Justus Liebig University, Heinrich-Buff-Ring 26-32, D-35392 Giessen, Germany; 3 Biometry and Population Genetics, Institute of Agronomy and Plant Breeding II, Interdisciplinary Research Center for Biosystems, Land Use and Nutrition (IFZ), Justus Liebig University, Heinrich-Buff-Ring 26-32, D-35392 Giessen, Germany

**Keywords:** Older adults, Folate, Cobalamin, Creatinine, Dietary intake, Longitudinal study

## Abstract

**Background:**

Older people are reported to be prone to imbalances between cobalamin and folate status with possible adverse effects on health. This longitudinal study investigates dynamics and interactions of cobalamin and folate status in a cohort of community-dwelling older adults by considering possible influencing factors.

**Methods:**

In total, 332 subjects ≥ 60 years were investigated over a mean observation period of 12 years. Data collection included serum cobalamin, folate and creatinine, dietary intakes of cobalamin, folate and alcohol, use of supplements, body composition, smoking behavior, and diseases. Linear mixed-effects models with repeated measurements were used to investigate the influence of variables on serum cobalamin and folate.

**Results:**

At baseline, median cobalamin intake exceeded the dietary reference value (DRV), while median folate intake was considerably below DRV. In most subjects, serum concentrations of both vitamins were within reference ranges. For serum cobalamin, apart from supplement use (Parameter estimate [95% confidence interval]: 130.17 [53.32, 207.01]), the main positive predictor was serum folate (4.63 [2.64, 6.62]). For serum folate, serum creatinine (10.85 [4.85, 16.86]), use of supplements (7.86 [5.05, 10.67]), serum cobalamin (0.01 [< 0.01, 0.01]), and dietary folate intake (0.02 [0.01, 0.03]) were positive predictors. No main effects of age, sex, body composition, alcohol intake or smoking were found after adjusting for simultaneous inference.

**Conclusions:**

Advancing age, per se, is no risk factor for a decline in serum concentrations of cobalamin or folate in subjects ≥ 60 years. Suboptimal folate intake may limit the function of folate regarding the supply of methyl groups for methylation of cobalamin and subsequent creatine biosynthesis. The positive association of serum creatinine with folate deserves further exploration with regard to its possible relevance for maintaining energy dependent functional integrity in the course of ageing.

## Highlights

- Advancing age is per se no risk factor for a decline in serum cobalamin and folate
- Serum cobalamin and folate are positively interlinked
- Dietary cobalamin intake above reference value has no impact on serum cobalamin
- Serum creatinine is independently associated with serum folate

## Background

There is a continued increase in life expectancy, which, however, is accompanied by an increased prevalence of age-related chronic diseases in the advanced ages. Several geriatric symptoms have been associated with low cobalamin and/or low folate status, such as cognitive impairment [1, 2], Parkinson’s disease [3], sarcopenia [4] and frailty [5]. However, the causal relevance of these associations remains to be elucidated. An emerging focus is on the role of B-vitamins on epigenetic modification, such as DNA methylation, as an underlying mechanism in the pathogenesis of age-related diseases, including cancer, cardiovascular and neurological diseases [6–9]. DNA methylation requires the donation of methyl groups in which folate and cobalamin play a major role [9]. Folate and cobalamin interact with each other in the methionine cycle via the remethylation of homocysteine to methionine [10]. This process is important for folate retention, DNA synthesis and subsequent production of S-adenosyl-L-methionine (SAM) [10, 11]. The latter is required for the methylation of various substrates in cellular metabolism [11]. It has been estimated that about 40% of all SAM derived methyl groups are used for the synthesis of creatine, which plays a complex role in energy metabolism [12, 13]. So far, human studies considering possible interrelations between cobalamin and folate status in conjunction with creatine levels are hardly available.

According to epidemiological studies, an imbalance of folate and cobalamin status may negatively affect health status in older adults [14]. It is well known that cobalamin deficiency can cause functional folate deficiency [10, 15]. However, it is not known which possible mechanisms could explain the positive association between blood concentrations of both vitamins noted in some cross-sectional studies [16–20] and also found by our group [21].

Thus, more knowledge on the interdependency of folate and cobalamin status and possible influencing factors is important for deriving conclusions on the impact of these two vitamins on age-related diseases and corresponding preventive measures. Although older individuals more often have an inferior cobalamin status [22], at present, there is little evidence for an age-related deterioration in either folate or cobalamin status under the condition of adequate intake and intact absorption mechanisms. However, longitudinal studies are needed to draw conclusions on age-related alterations in either folate or cobalamin status that are independent of other influencing factors. Against this background, the present study investigates dynamics and interactions of folate and cobalamin status in a community-dwelling older cohort over a mean follow-up period of 12 years using multiple assessments. We also explore, whether folate and/or cobalamin status are possibly related to serum creatinine, which besides being used as a marker of kidney function serves as a proxy for total body pool of creatine [23].

## Subjects and methods

### Study cohort

The present investigation is based on the longitudinal study on nutrition and health status of senior citizens in Giessen (GISELA study), Germany, which is a prospective cohort study initiated in 1994 and completed in 2014. Participants were recruited by physicians, notices, senior citizens’ meetings, advertisements in local newspapers and through individuals, who had already participated. Eligibility criteria were an age of at least 60 years, physical mobility and resident in Giessen or surrounding areas. The investigations took place at the Institute of Nutritional Science in Giessen from July to October at annual intervals between 1994 and 1998 and since 1998 at biannual intervals. Subjects joined the study until 2006.

Overall, 587 subjects participated in the GISELA study. The assessed parameters varied among follow-ups and not all subjects took part in each follow-up. For the present analysis, data from the follow-ups in 1997, 1998, 2002, 2006, 2010, 2012 and 2014 were analyzed because duplicate measurements on both serum cobalamin and folate were available. Records were in the first instance excluded because of non-fasting state (*n* = 12) followed by the exclusion of records with missing data for serum cobalamin, serum folate, body composition, dietary intake and use of supplements, respectively (*n* = 285). Finally, only subjects with at least three complete data records on relevant factors were considered, resulting in a study sample of 332 subjects with 1556 records, 233 women and 99 men. A flow chart of the numbers and reasons for excluding subjects is presented in the online supporting material (Additional file 1: Figure S1). The final number of subjects differed by follow-up year: 1997 (*n* = 243), 1998 (*n* = 254), 2002 (*n* = 310), 2006 (*n* = 251), 2010 (*n* = 185), 2012 (*n* = 176) and 2014 (*n* = 137). Of the final cohort, 89, 73, 71, 51 and 48 subjects participated in 3, 4, 5, 6 and 7 follow-ups, respectively. The mean follow-up time was 12 years.

### Anthropometric data and body composition

Body mass index (BMI) was calculated from measured body height and body mass. Body composition was assessed by bioelectrical impedance analysis (Akern-RJL BIA 101/S, Data Input, Frankfurt, Germany, frequency: 50 kHz) as described previously [24].

### Assessment of dietary intake, use of supplements, lifestyle factors and diseases

Intakes of food and beverages were determined by a three-day estimated dietary record, which was developed and validated for the GISELA study and included 146 food items [25]. The German Nutrient Database version 3.02 (Max Rubner-Institute, Karlsruhe, Germany) was used to calculate dietary intake levels of cobalamin, folate and alcohol. The dietary assessment did not include intake by supplements. Data on supplement use and smoking behavior were collected via self-administered questionnaires. The use of vitamin B and multi-vitamin supplements was coded as dummy variable into users and non-users. Smoking behavior was classified as dummy variable in never-smokers and current/ex-smokers. Subjects who reported to be current/ex-smokers at any time point in the study were considered as current/ex-smokers throughout the study. Subjects with missing values for smoking behavior at any time point were considered as non-smokers throughout the study if they reported to be never-smokers at all other available time points. Participants reported diseases diagnosed by their physicians using a questionnaire including predefined disease categories and the option to complement this list.

### Biochemical analyses

Blood samples were collected between 7:00 a.m. and 11:00 a.m. after an overnight fast. After coagulation of blood samples, samples were immediately centrifuged and serum aliquots were stored at about −70 °C until analysis.

Serum cobalamin and folate were assessed in duplicates by SimulTRAC-SNB radio assay kit (former ICN Biomedicals, later MP Biomedicals, Eschwege, Germany), which represents a competitive protein binding assay. Serum cobalamin and folate measurements were both available for the follow-ups in 1997, 1998, 2002, 2006, 2010, 2012 and 2014. The day-to-day variation was < 13.5% for serum cobalamin (except for 1997: 22.9%) and < 10.6% for serum folate. Serum creatinine was measured in 2002, 2006, 2008, 2010, 2012 and 2014 by a kinetic color reaction according to Jaffé [26]. The day-to-day variation for serum creatinine was ≤ 3.5%. For the present investigation, serum creatinine measurements from 2002, 2006, 2010, 2012 and 2014 were used, because for 2008, no data on serum cobalamin and serum folate were available.

Serum cobalamin ≤ 148 pmol/L and serum folate < 7 nmol/L were used as cut-off values to identify cobalamin- and folate-deficient subjects, respectively [11, 27]. In addition, alternative cut-off values of ≤ 221 pmol/L for serum cobalamin and < 10 nmol/L for serum folate were applied to identify suboptimal supplied subjects [28, 29].

### Statistical analysis

Statistical analyses are of exploratory nature and were performed with the software R, version 3.6.3. Results are considered statistically significant when *P* < 0.050.

Because of the presence of non-normally distributed data, tested by Shapiro-Wilk test, non-parametric tests were performed to investigate descriptive characteristics. Metric data were expressed as median and 25 and 75% quartiles, if not stated otherwise.

Linear mixed-effects models with repeated measurements were used in order to investigate the influence of all relevant variables on serum cobalamin and serum folate, respectively. Analyses were carried out with the nlme package [30], version 3.1–145, and multcomp [31], version 1.4–13, with a continuous AR (1) correlation structure. We used different models to identify the predictors of serum cobalamin and serum folate. The term ‘predictors’ refers to the investigated main effects, which showed a significant association with the respective dependent variable. Each model considered age and subject as random effects, thus allowed random slopes and intercepts. The **first model** included only age as fixed effect and consequently represents the unadjusted model. The **second model** investigated besides age, sex, use of vitamin B/multi-vitamin supplements, absolute fat-free mass (FFM), smoking behavior, dietary intake of cobalamin, folate and alcohol and serum folate or serum cobalamin as main effects and potential effect modifications by age and/or sex with regard to serum folate or serum cobalamin, use of supplements and cobalamin or folate intake by studying pairwise interactions. To study the impact of body composition more in detail, absolute FFM was replaced by relative FFM, absolute FM and BMI, respectively. The **third model** examined the influence of serum creatinine on serum cobalamin and serum folate. Since serum creatinine measurements were only available for five of the seven follow-ups, a sub-sample was used. After removing subjects with less than three follow-ups with complete data records, 202 subjects remained for this analysis. In model 3, besides serum creatinine all independent variables of model 2 were included. The **fourth model** based on model 3, but excluded records of subjects reporting the use of vitamin B/multi-vitamin supplements, leaving 161 subjects, who participated in at least three follow-ups. QQ-normal plots for residuals of the fitted models indicated deviations from normal distribution at both ends and considerable heavier tails. Therefore, analyses were repeated with logarithmically transformed (log_10_) serum cobalamin and serum folate concentrations and without records with outlying residuals (see sensitivity analysis below). The presented *P* values of the linear mixed-effects models were adjusted for simultaneous inference [31].

The following sensitivity analyses were performed:

1. Because of the small time line between 1997 and 1998 and the higher coefficient of variation for serum cobalamin in 1997 compared to other follow-ups, models 1 and 2 were repeated without data from 1997 to investigate the robustness of the results. By doing this, the sample size was reduced to 263 subjects. Models 3 and 4 were not repeated as these models included only follow-ups between 2002 and 2014 due to the inclusion of serum creatinine.
2. To study the robustness of the association between serum folate and serum cobalamin, models 2 to 4 were rerun after excluding subjects with a reported lifetime diagnosis of cancer (*n* = 71 based on the sample size of 332 subjects) or inflammatory bowel disease (*n* = 11 based on the sample size of 332 subjects) or records with serum cobalamin concentrations above 1000 pmol/L (44 records based on the sample size of 332 subjects), as these values might indicate the use of high doses of supplements or the presence of serious chronic diseases. By doing this, the sample sizes were reduced to 246 (model 2), 148 (model 3) and 124 (model 4) subjects, respectively. Furthermore, to obtain normally distributed residuals, serum cobalamin and serum folate were log_10_ transformed and records with outlying residuals for the respective model were excluded. Thus, the final sample size differed across models for serum cobalamin and serum folate.

For interpretation of the models, Akaike’s information criterion (AIC), the residual variance and the Pearson product-moment correlation coefficient between observed and predicted serum cobalamin or serum folate values were used. Estimation of the variance components was done with restricted maximum likelihood (REML). Metric independent variables were mean centered to facilitate the interpretation of the results and to counteract multicollinearity.

## Results

### Baseline characteristics of the study population

Table 1 summarizes the baseline characteristics of the study subjects, i.e. their first available measurements. Women had higher serum cobalamin, showed lower FFM, energy intake, cobalamin intake and alcohol intake and reported less often to be current or ex-smoker compared to men. Serum folate, folate intake, age, BMI, serum creatinine and use of vitamin B/multi-vitamin supplements did not differ by sex.

**Table 1:** Baseline characteristics of the study subjects^a^

	Total (*n* = 332)	Women (*n* = 233)	Men (*n* = 99)	*P*^b^
Serum cobalamin (pmol/L)	266.8 (207.6, 376.8)	272.4 (216.3, 382.2)	248.7 (186.9, 356.0)	0.036
Serum folate (nmol/L)	19.0 (15.0, 23.8)	19.2 (15.1, 24.9)	18.3 (14.3, 22.3)	0.133
Age (years)	68 (64, 71)	68 (63, 72)	67 (64, 70)	0.526
Body mass index (kg/m^2^)	26.2 (24.1, 28.6)	26.3 (23.9, 28.7)	26.1 (24.6, 28.6)	0.840
Fat mass (kg)	26.4 (22.1, 32.5)	28.2 (23.4, 34.1)	22.9 (19.0, 28.4)	< 0.001
Fat-free mass (kg)	41.7 (38.7, 51.9)	39.6 (37.8, 42.3)	54.8 (52.7, 58.5)	< 0.001
Fat-free mass (%)	61.6 (56.3, 67.6)	58.4 (54.8, 62.4)	70.5 (66.7, 74.1)	< 0.001
Energy intake (kcal/d)	2012 (1676, 2369)	1919 (1607, 2248)	2262 (1895, 2609)	< 0.001
Cobalamin intake (μg/d)	5.3 (4.0, 7.1)	5.1 (3.8, 6.9)	5.7 (4.6, 7.6)	0.009
Folate intake (μg/d)	236.0 (194.6, 290.1)	235.3 (186.3, 288.8)	248.1 (200.3, 304.8)	0.153
Alcohol intake (g/d)	3.7 (0.3, 11.2)	3.3 (0.3, 7.6)	11.2 (0.4, 20.0)	< 0.001
Serum creatinine (mg/dL) ^c^	0.9 (0.9, 1.0)	0.9 (0.9, 1.0)	1.0 (0.9, 1.1)	0.131
Vitamin B/multi-vitamin supplement users	106 (31.9)	78 (33.5)	28 (28.3)	0.424
Current/ex-smokers	135 (40.7)	62 (26.6)	73 (73.7)	< 0.001

^a^Metric variables are expressed as median and 25 and 75% quartile, whereas categorical variables are expressed as absolute and relative frequencies

^b^*P* values for comparison between sexes by Wilcoxon-Mann-Whitney test and Chi-squared test

^c^In the baseline data set, only 58 subjects (47 females and 11 males) had serum creatinine measurements, because serum creatinine was first measured in 2002 and the majority of the subjects entered the study before 2002

At baseline, 22 (9%) and 64 (27%) females and 16 (16%) and 40 (40%) males had serum cobalamin ≤ 148 pmol/L and ≤ 221 pmol/L, while 66 females (28%) and 15 males (15%) had a cobalamin intake < 4 μg/d, respectively. Concerning folate, 2 (1%) and 20 (9%) females and 1 (1%) male and 6 (6%) males showed serum folate < 7 nmol/L and < 10 nmol/L, whereas 181 females (78%) and 73 males (74%) had a folate intake < 300 μg/d at baseline, respectively. Users of vitamin B/multi-vitamin supplements had higher serum cobalamin (median: 296.8 vs. 259.8 pmol/L, *P* < 0.050) and serum folate (median: 22.3 vs. 18.1 nmol/L, *P* < 0.050) than non-users.

### Longitudinal analysis on predictors of serum cobalamin and serum folate

Without consideration of other fixed effects, a positive influence of age on serum cobalamin (parameter estimate [95% CI] = 4.43 [0.99, 7.87]; *P* < 0.050; AIC = 22,776) and serum folate (0.27 [0.13, 0.42]; *P* < 0.050; AIC = 12,684) was found. Following logarithmical transformation of the dependent variable, the positive age effect remained significant (both *P* < 0.050).

Concerning serum cobalamin, the results of the linear mixed-effects models are summarized in Table 2. In model 2, serum folate and use of vitamin B/multi-vitamin supplements were besides pairwise interactions between serum folate and age as well as serum folate and sex positive predictors of serum cobalamin after adjusting for simultaneous inference. No main effects of age, absolute FFM, dietary intake, smoking and sex were found. When absolute FFM was replaced by relative FFM, absolute FM or BMI, the results remained approximately unchanged (Additional file 2: Table S1).

**Table 2:** Longitudinal predictors of serum cobalamin using linear mixed-effects models^a^

	Model 2 with total sample (*n* = 332)		Model 3 with serum creatinine (*n* = 202)		Model 4 without users of vitamin B/multi-vitamin supplements (*n* = 161)	
	PE [95% CI]	*P*^b^	PE [95% CI]	*P*^b^	PE [95% CI]	*P*^b^
Intercept	316.79 [271.75, 361.82]	< 0.001	312.98 [247.58, 378.38]	< 0.001	297.65 [240.62, 354.68]	< 0.001
Serum folate (nmol/L)	3.52 [2.03, 5.02]	< 0.001	4.63 [2.64, 6.62]	< 0.001	4.08 [2.19, 5.98]	< 0.001
Vitamin B/multi-vitamin supplementation	96.86 [50.66, 143.06]	< 0.001	130.17 [53.32, 207.01]	0.014		
Age (years)	2.31 [−2.12, 6.73]	0.991	2.95 [−4.08, 9.98]	0.999	3.81 [−2.92, 10.55]	0.975
Fat-free mass (kg)	−1.83 [−7.21, 3.55]	1.000	−2.80 [−10.09, 4.49]	1.000	−1.84 [−7.45, 3.78]	1.000
Cobalamin intake (μg/d)	2.16 [−4.25, 8.58]	1.000	−3.01 [−12.06, 6.04]	1.000	1.48 [−5.98, 8.94]	1.000
Folate intake (μg/d)	−0.10 [−0.33, 0.12]	0.998	0.01 [−0.36, 0.38]	1.000	−0.17 [−0.49, 0.15]	0.986
Alcohol intake (g/d)	−0.79 [−2.80, 1.23]	1.000	−0.18 [−3.39, 3.02]	1.000	−0.11 [−2.36, 2.14]	1.000
Past/current smoking	6.30 [−48.48, 61.08]	1.000	13.63 [−67.30, 94.56]	1.000	−22.00 [−86.54, 42.55]	1.000
Male sex	3.38 [−100.76, 107.51]	1.000	11.32 [−137.29, 159.92]	1.000	25.24 [−98.84, 149.33]	1.000
Age x serum folate	0.38 [0.22, 0.54]	< 0.001	0.53 [0.28, 0.78]	< 0.001	0.76 [0.50, 1.02]	< 0.001
Age x supplementation	4.13 [−2.45, 10.71]	0.959	4.03 [−7.25, 15.31]	1.000		
Age x cobalamin intake	−0.50 [−1.17, 0.17]	0.866	−0.10 [−1.13, 0.93]	1.000	0.27 [−0.63, 1.16]	1.000
Age x sex	0.17 [−7.16, 7.50]	1.000	3.02 [−8.45, 14.49]	1.000	0.13 [−11.57, 11.82]	1.000
Sex x serum folate	7.56 [4.93, 10.18]	< 0.001	7.15 [3.60, 10.71]	0.001	8.20 [5.11, 11.29]	< 0.001
Serum creatinine (mg/dL)			−3.64 [−169.46, 162.19]	1.000	18.50 [−117.87, 154.86]	1.000
Model fit ^c^	0.705		0.722		0.821	
Residual standard deviation	312.06		345.16		226.93	
AIC	22,561		12,630		9014	

*PE* Parameter estimate, *95% CI* 95% confidence interval, *AIC* Akaike’s information criterion

^a^Data represent the results of the linear mixed-effects models including serum cobalamin concentrations as dependent variable, random effects of age and subject and centered metric independent variables. Model 2 considered as fixed effects: serum folate, vitamin B/multi-vitamin supplementation, age, absolute fat-free mass, cobalamin intake, folate intake, alcohol intake, smoking, sex and effect modifications by sex and age. Model 3 based on model 2 and considered serum creatinine as additional fixed effect. Model 4 based on model 3 but excluded records, in which use of vitamin B/multi-vitamin supplements was reported

^b^Denotes *P* values after adjusting for simultaneous inference

^c^Correlation between observed and predicted serum cobalamin concentrations

Concerning serum folate, the results of the linear mixed-effects models are summarized in Table 3. In model 2, serum cobalamin, use of vitamin B/multi-vitamin supplements and folate intake were significant positive predictors of serum folate besides a pairwise interaction between sex and serum cobalamin after adjusting for simultaneous inference. No main effects of age, absolute FFM, cobalamin intake, alcohol intake, smoking and sex were found. When absolute FFM was replaced by relative FFM, absolute FM or BMI, these results were unchanged (Additional file 2: Table S2).

**Table 3:** Longitudinal predictors of serum folate using linear mixed-effects models^a^

	Model 2 with total sample (*n* = 332)		Model 3 with serum creatinine (*n* = 202)		Model 4 without users of vitamin B/multi-vitamin supplements (*n* = 161)	
	PE [95% CI]	*P*^*b*^	PE [95% CI]	*P*^*b*^	PE [95% CI]	*P*^*b*^
Intercept	22.24 [20.52, 23.95]	< 0.001	22.18 [19.78, 24.57]	< 0.001	22.27 [19.94, 24.60]	< 0.001
Serum cobalamin (pmol/L)	0.006 [0.003, 0.008]	< 0.001	0.007 [0.004, 0.010]	< 0.001	0.011 [0.006, 0.017]	0.001
Vitamin B/multi-vitamin supplementation	6.52 [4.80, 8.24]	< 0.001	7.86 [5.05, 10.67]	< 0.001		
Age (years)	0.21 [0.03, 0.38]	0.255	0.12 [−0.15, 0.39]	0.999	0.08 [−0.16, 0.33]	1.000
Fat-free mass (kg)	−0.13 [−0.34, 0.09]	0.973	−0.28 [−0.56, 0.01]	0.568	−0.02 [−0.31, 0.27]	1.000
Cobalamin intake (μg/d)	−0.16 [−0.40, 0.08]	0.923	−0.11 [−0.44, 0.22]	1.000	0.10 [−0.25, 0.45]	1.000
Folate intake (μg/d)	0.02 [0.01, 0.03]	< 0.001	0.02 [0.01, 0.03]	0.083	0.02 [0.01, 0.04]	0.018
Alcohol intake (g/d)	0.01 [−0.07, 0.09]	1.000	0.03 [−0.09, 0.16]	1.000	0.03 [−0.08, 0.15]	1.000
Past/current smoking	−1.08 [−3.21, 1.04]	0.993	−0.17 [−3.21, 2.87]	1.000	1.45 [−1.66, 4.55]	0.994
Male sex	0.96 [−3.06, 4.97]	1.000	1.68 [−3.92, 7.29]	1.000	−2.64 [−8.33, 3.05]	0.995
Age x serum cobalamin	< 0.01 [> −0.01, < 0.01]	0.915	> −0.01 [> −0.01, < 0.01]	1.000	> −0.01 [> −0.01, > −0.01]	0.045
Age x supplementation	0.25 [< 0.01, 0.50]	0.474	0.06 [−0.36, 0.48]	1.000		
Age x folate intake	< 0.01 [> −0.01, < 0.01]	0.958	< 0.01 [> −0.01, < 0.01]	0.797	> −0.01 [> −0.01, < 0.01]	1.000
Age x sex	−0.11 [−0.40, 0.19]	1.000	−0.11 [−0.57, 0.34]	1.000	−0.04 [−0.47, 0.39]	1.000
Sex x serum cobalamin	0.01 [0.01, 0.02]	< 0.001	0.01 [0.01, 0.02]	< 0.001	0.02 [0.01, 0.02]	< 0.001
Serum creatinine (mg/dL)			10.85 [4.85, 16.86]	0.006	7.69 [1.92, 13.46]	0.107
Model fit ^c^	0.620		0.668		0.552	
Residual standard deviation	13.30		14.07		12.60	
AIC	12,534		7130		5172	

*PE* Parameter estimate, *95% CI* 95% confidence interval, *AIC* Akaike’s information criterion

^a^Data represent the results of the linear mixed-effects models including serum folate concentrations as dependent variable, random effects of age and subject and centered metric independent variables. Model 2 considered as fixed effects: serum cobalamin, vitamin B/multi-vitamin supplementation, age, absolute fat-free mass, cobalamin intake, folate intake, alcohol intake, smoking, sex and effect modifications by sex and age. Model 3 based on model 2 and considered serum creatinine as additional fixed effect. Model 4 based on model 3 but excluded records, in which use of vitamin B/multi-vitamin supplements was reported

^b^Denotes *P* values after adjusting for simultaneous inference

^c^Correlation between observed and predicted serum folate concentrations

### Longitudinal analysis on predictors of serum cobalamin and serum folate with serum creatinine as additional main effect

The additional inclusion of serum creatinine as main effect did not substantially modify the results for serum cobalamin (Table 2, model 3). In contrast, serum creatinine was an independent positive predictor of serum folate and the main effect of folate intake on serum folate became borderline significant (Table 3, model 3).

### Longitudinal analysis on predictors of serum cobalamin and serum folate in non-users of vitamin B/multi-vitamin supplements

After excluding records in which use of vitamin B/multi-vitamin supplements were reported, serum folate and pairwise interactions between serum folate and age as well as serum folate and sex were still positive predictors of serum cobalamin (Table 2, model 4). With regard to serum folate, significant positive predictors were serum cobalamin, folate intake and the pairwise interaction between sex and serum cobalamin, while the interaction between age and serum cobalamin was inversely associated with serum folate (Table 3, model 4).

### Sensitivity analyses

When models 1 and 2 were repeated without data from 1997, the overall results remained unchanged for serum cobalamin and serum folate (data not shown).

After excluding subjects with lifetime diagnosis of cancer or inflammatory bowel disease and records with serum cobalamin concentrations above 1000 pmol/L or residuals outside of the assumed normal distribution, serum folate was still in each model positively associated with serum cobalamin and vice versa (*P* < 0.001). For serum cobalamin, serum folate was the only main effect, which was identified as significant positive predictor in all models after adjusting for simultaneous inference (Additional file 2: Table S3). The former main effect of supplement usage on serum cobalamin vanished. As regards serum folate, all main effects remained significant (Additional file 2: Table S4). Furthermore, the positive main effect of folate intake in model 3 and serum creatinine in model 4 reached significance even after adjusting for multiple testing, while the interaction between sex and serum cobalamin abolished. The unadjusted and adjusted association of serum cobalamin with serum folate is illustrated in the Additional file 1: Figure S2.

### Illustration of the model prediction

**Fig. 1:**
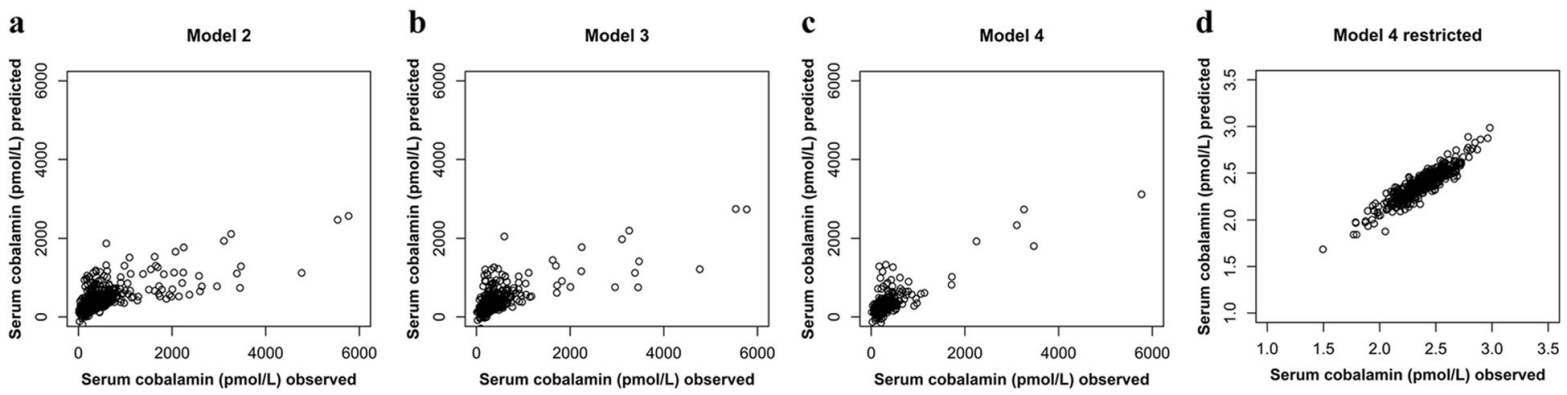
Association between observed serum cobalamin concentrations and values predicted by the respective linear mixed-effects model. This figure illustrates the model fits of models 2, 3 and 4 as well as the model fit of the restricted model 4 applied in sensitivity analysis. Model fit was 0.705, 0.722, 0.821 and 0.916, respectively. **a** Model 2 with serum cobalamin as dependent variable and fixed effects for serum folate, vitamin B/multi-vitamin supplementation, age, absolute fat-free mass, cobalamin intake, folate intake, alcohol intake, smoking, sex and effect modifications by sex and age (*n* = 332); **b** Model 3 based on model 2 but is restricted to subjects with creatinine measurements and included serum creatinine as additional fixed effect (*n* = 202); **c** Model 4 based on model 3 but excluded records with use of vitamin B/multi-vitamin supplements (*n* = 161); and (**d**) Model 4 without subjects with lifetime diagnosis of cancer/inflammatory bowel disease and records with serum cobalamin > 1000 pmol/L or residuals outside of the assumed normal distribution (*n* = 121); serum cobalamin concentrations were logarithmically transformed for normalization purposes

**Fig. 2:**
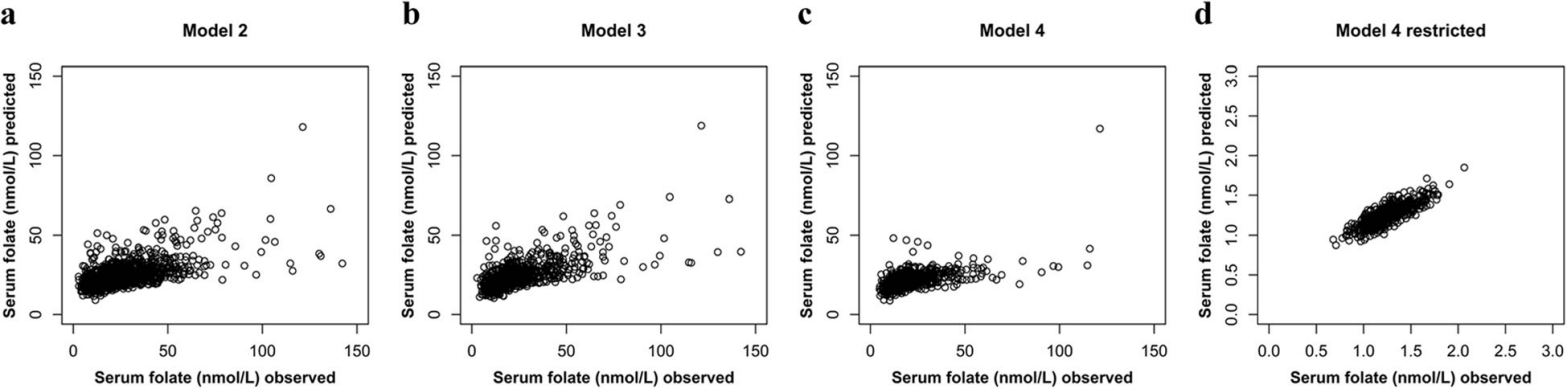
Association between observed serum folate concentrations and values predicted by the respective linear mixed-effects model. This figure illustrates the model fits of models 2, 3 and 4 as well as the model fit of the restricted model 4 applied in sensitivity analysis. Model fit was 0.620, 0.668, 0.552 and 0.834, respectively. **a** Model 2 with serum folate as dependent variable and fixed effects for serum cobalamin, vitamin B/multi-vitamin supplementation, age, absolute fat-free mass, cobalamin intake, folate intake, alcohol intake, smoking, sex and effect modifications by sex and age (*n* = 332); **b** Model 3 based on model 2 but is restricted to subjects with creatinine measurements and included serum creatinine as additional fixed effect (*n* = 202); **c** Model 4 based on model 3 but excluded records with use of vitamin B/multi-vitamin supplements (*n* = 161); and (**d**) Model 4 without subjects with lifetime diagnosis of cancer/inflammatory bowel disease and records with serum cobalamin > 1000 pmol/L or residuals outside of the assumed normal distribution (*n* = 122); serum folate concentrations were logarithmically transformed for normalization purposes

The associations between observed and predicted serum cobalamin and serum folate based on the different linear mixed-effects models are presented in Figs. 1 and 2, respectively. The model fit ranged between 0.6 and 0.9. For serum folate, model fit was generally lower than for serum cobalamin. After excluding subjects with lifetime diagnosis of cancer or inflammatory bowel disease and records with serum cobalamin concentrations above 1000 pmol/L or residuals outside of the assumed normal distribution, considerably lower scattering and an improved model fit were found for both serum cobalamin and serum folate.

## Discussion

The unique feature of this study is a prospective mean observation period of 12 years on the dynamics and interactions of cobalamin and folate status in community-dwelling older subjects by consideration of relevant covariates. The main findings are the following: For serum cobalamin, apart from the use of supplements, the main positive predictor is serum folate. For serum folate, serum creatinine, use of supplements, serum cobalamin, and dietary folate intake are positive predictors. Effect modifications with regard to age and sex are evident for both vitamins.

The baseline cobalamin and folate intakes of our subjects are comparable to age-matched subjects in the German Nationwide Nutrition Survey II [32] and within the range of cobalamin [27] and folate [11] intakes across European countries. Median intakes of cobalamin exceed German [28] and European [27] dietary reference values (DRVs) of 4 μg/d, while median folate intakes are considerably below DRVs of 300 μg dietary folate equivalent (DFE)/d [29] or 330 μg DFE/d [11]. Nevertheless, in most subjects, serum concentrations of both vitamins are within accepted reference ranges of > 148–221 pmolL for cobalamin [27, 28] and of ≥ 7–10 nmol/L for folate [11, 29] and are comparable to other European populations in this age range [11, 27]. However, about one third of the subjects had serum cobalamin concentrations below the alternative cut-off value for defining adequate serum cobalamin concentrations and the study cohort shows considerably lower folate status compared to the U.S. population aged > 60 years [33, 34], where folate intakes are much higher due to national fortification policies [34, 35].

Our longitudinal analyses do not reveal a resilient association of dietary cobalamin intake with serum cobalamin, possibly because median dietary cobalamin intake is at a level where intestinal absorption capacity via intrinsic factor [36] and serum cobalamin level off [37]. However, we cannot exclude that other factors are responsible for the missing association, such as undiagnosed diseases, which affect absorption or metabolism of cobalamin [38, 39]. The use of supplements was associated with higher serum cobalamin concentrations, but the association vanished in sensitivity analysis, what may be explained by the fact that high-dosed cobalamin supplements can be absorbed by passive diffusion [40]. In contrast to cobalamin, serum folate is associated with its intake, both via diet and supplements. This may be because dietary folate intake is below DRVs, such that saturation of absorption or plasma pool may not be achieved.

In our longitudinal analysis, serum folate is a main positive predictor of serum cobalamin what confirms the findings of our previous cross-sectional analysis [21]. As indicated in the Additional file 1: Figure S2, the adjusted association between serum cobalamin and serum folate seems not to level off even at high concentrations. The reciprocal positive association of serum folate with serum cobalamin emphasizes the close interrelationship between these two vitamins, which presumably depends on the provision and need of methyl groups [10].

Interestingly, our data show a pronounced positive association of serum creatinine with serum folate but not with serum cobalamin. Likewise, a cross-sectional study in older Swiss subjects found no associations between estimated glomerular filtration rate (eGFR) and serum cobalamin or holotranscobalamin, but a positive association between eGFR and serum folate [41]. Creatinine concentrations depend in particular on the breakdown of phosphocreatine in muscle, use of creatine supplements and dietary meat intake [42]. Thus, the observed association may be attributed to other causes than a decline in kidney function. In literature, positive cross-sectional associations between urinary creatinine concentrations and plasma folate and cobalamin were reported and a role of folate and cobalamin in creatine biosynthesis was suspected [43]. The association between serum folate and creatinine could depend on the function of methyl-tetrahydrofolate in the methylation of methionine to SAM. SAM is the methyl donor for the majority of methyl-transferases, among which guanidinoacetate methyltransferase has been reported to consume approximately 40% of all SAM-derived methyl groups for the synthesis of creatine [12, 13]. Serum creatinine, though mostly used as marker of kidney function, is a proxy for total creatine pool from which it is formed in a constant overall conversion rate of about 1.7% per day [23]. Our finding that folate and creatinine are closely interrelated is in line with early observations in rhesus monkeys [44] and mice [45] demonstrating that folic acid is involved in the formation of creatine. Our data do not allow to conclude whether folate impacts cobalamin or creatinine or vice versa. However, in vitro and in vivo studies in mice led to the conclusion that creatine formation is primarily influenced by folic acid and that cobalamin exerts its effect by promoting better utilization of folic acid at low levels [45]. This in mind, we additionally investigated serum creatinine as dependent variable and serum folate and cobalamin as independent variables by controlling for sex, age, FFM, dietary intake, supplement usage, alcohol consumption and smoking behavior. In this analysis, serum folate (*P* < 0.001) besides age (*P* < 0.001) was an independent positive predictor for log_10_ serum creatinine even after adjusting for multiple testing and excluding records with outlying residuals (data not shown). As to the question why folate but not cobalamin is associated with creatinine, several possible causes could explain this finding: 1) the biomarker serum cobalamin is not sensitive enough to reflect the association between cobalamin and creatinine (as serum cobalamin is carrying both metabolic active and inactive cobalamins), 2) folate rather than cobalamin is a limiting nutrient for the synthesis of creatine and 3) folate intake was consistently associated with serum folate and predominantly in a range considered as insufficient, while dietary cobalamin did not affect serum cobalamin. Thus, the low folate intake may limit the function of folate regarding the supply of methyl groups for methylation of cobalamin and subsequent creatine biosynthesis, even though the low folate intake was still sufficient to keep serum folate for most individuals within the current reference range. Creatine plays a role in cellular energy metabolism by being involved in energy transport and replenishment of adenosine triphosphate under anaerobic conditions [46]. The results in our study may therefore suggest a relationship between serum folate and energy metabolism. Impaired bioenergetics in aged tissues such as muscle and brain have been linked to declines in muscle strength and physical and cognitive performance [46–49]. However, whether or to which extent folate status may play a role or interfere in such processes is presently unknown.

With regard to the impact of age on biomarkers of cobalamin and folate status the results from cross-sectional studies are inconsistent; for both vitamins negative, positive or no correlations were observed [16, 21, 33, 41, 50, 51]. For example, in a study on subjects aged ≥ 60 years, no age-related differences in serum cobalamin or red blood cell folate were found, while serum folate decreased across age groups [41]. In a study on subjects aged between 35 and 80 years, serum cobalamin decreased and serum folate remained stable across age groups [16]. In NHANES, serum cobalamin and folate increased with increasing age in subjects ≥ 20 years even after adjusting for cofactors [51]. For investigating effects of age, longitudinal surveys are most appropriate as these enable to assess changes during individual aging by considering potential influencing factors [52]. We are not aware of any other longitudinal study that investigated the independent effect of age on cobalamin and folate status in an elderly cohort by using multiple follow-ups. In our longitudinal analysis, a positive effect of age on serum cobalamin or serum folate is only found when covariables are not considered. Nevertheless, advancing age seems to enhance the effect of serum folate on serum cobalamin, irrespectively of the level of adjustment. Furthermore, male sex is associated with a stronger positive effect of serum cobalamin on serum folate and vice versa, but sex shows no direct influence on the absolute serum cobalamin and folate concentrations after multiple adjustments. Because in our study advancing age is also associated with higher serum creatinine concentrations, one may speculate that age- and sex-specific characteristics may be involved in the metabolism/transfer of methyl groups. In sensitivity analyses, however, effect modifications by sex and age mostly vanished.

Some cross-sectional studies which did not focus on subjects ≥ 60 years have reported associations of smoking behavior with serum folate [33, 51, 53] or serum cobalamin [18, 51] and of BMI with serum folate [33, 54] or serum cobalamin [51], respectively. In addition, alcohol consumption was associated with circulating B-vitamin concentrations in previous studies [18, 33, 51]. We therefore included these parameters in our analyses. However, alcohol intake, smoking, BMI and body composition did not prove to be factors influencing serum cobalamin or serum folate after adjusting for simultaneous inference. These results are in line with findings from cross-sectional studies in older adults in which cobalamin and folate status were not associated with nutritional status and BMI [55] or smoking and alcohol intake [16].

Some limitations of our study should be considered. We used fasting serum concentrations of cobalamin and folate to assess status of these vitamins. Serum holotranscobalamin is recognized as the most specific biomarker for cobalamin status, but uniform cut-off values are lacking [27]. Nevertheless, comparable performances of holotranscobalamin and serum cobalamin concentrations were reported with respect to the screening of cobalamin-deficient subjects [56]. Serum folate is a sensitive marker of dietary intake [11] and correlates with red blood cell folate [19, 33]. Fasting serum folate has been suggested as preferred biomarker for folate status [19, 57]. We did not include genetic profiles or detailed data on diseases and use of medicines in our analyses. The main reasons for loss to follow-up in the GISELA study were sickness, no interest and death [unpublished results]. Those subjects of the GISELA study who were lost to follow-up or excluded due to missing data, were older and smaller, but were similar as regards body composition, energy intake and physical activity [unpublished results]. Thus, selection bias may have occurred, what may have led to an underestimation of associations. Finally, the sample consisted of volunteers and thus data may not be generalizable to community-dwelling subjects in Germany.

As strengths of our study, we want to point out the longitudinal design including multiple follow-ups over nearly two decades, the consideration of relevant predictors and interactions as well as consistent measurement conditions over the study period. We conducted sensitivity analyses, all of which corroborate the robustness of our main results. The model fits indicate a good to very good prediction of our models. Based on the unique features of the study design and elaborate statistical analysis of data, the main contribution of our results to existing knowledge is the demonstration of a sensitive metabolic interaction of serum cobalamin, folate and creatine/creatinine in the course of advancing age even in an overall well-nourished community-dwelling elderly cohort.

## Conclusions

This longitudinal study reveals that advancing age, per se, is no risk factor for a decline in serum concentrations of cobalamin or folate in community-dwelling subjects aged ≥ 60 years with cobalamin and folate status predominantly within reference ranges at baseline. Thus, an adequate cobalamin and folate status in early ageing may be advantageous for ensuring adequacy also in older ages. The results suggest a robust positive association between serum cobalamin and folate and vice versa under the condition that DRVs for cobalamin are met and folate intakes are below DRVs but still are sufficient to maintain serum folate within current reference range. Furthermore, serum folate is strongly associated with serum creatinine. Suboptimal folate intake therefore may limit the function of folate regarding the supply of methyl groups for methylation of cobalamin and subsequent creatine biosynthesis. As creatine has important functions in energy metabolism, the interaction of cobalamin and folate status with creatine deserves further exploration with regard to its possible relevance for maintaining energy dependent functional integrity in the course of ageing.

## Supplementary information

**Additional file 1: Figure S1.** Flow chart of the present investigation **Figure S2.** Association between serum cobalamin and logarithmically transformed serum folate before and after adjustments for covariables within linear mixed-effects model.

**Additional file 2: Table S1.** Predictors of serum cobalamin using linear mixed-effects models with BMI, relative FFM and absolute FM as main effects (*n* = 332) **Table S2.** Predictors of serum folate using linear mixed-effects models with BMI, relative FFM and absolute FM as main effects (*n* = 332) **Table S3.** Predictors of logarithmically transformed serum cobalamin using linear mixed-effects models without subjects with lifetime diagnosis of cancer/inflammatory bowel disease and records with serum cobalamin > 1000 pmol/L or with outlying residuals **Table S4.** Predictors of logarithmically transformed serum folate using linear mixed-effects models without subjects with lifetime diagnosis of cancer/inflammatory bowel disease and records with serum cobalamin > 1000 pmol/L or with outlying residuals.

## Data Availability

The datasets analyzed during the current study are available from the corresponding author on reasonable request.

## Abbreviations

95% CI: 95% Confidence interval
AIC: Akaike’s information criterion
BIA: Bioelectrical impedance analysis
BMI: Body mass index
EFSA: European Food Safety Authority
FFM: Fat-free mass
FM: Fat mass
GISELA: Longitudinal study on nutrition and health status of senior citizens in Giessen
REML: Restricted maximum likelihood
SAM: S-adenosyl-L-methionine
