# Supplementary material for: Dynamics and interactions of cobalamin and folate status during advanced aging – a longitudinal study in a community-dwelling cohort with multiple follow-ups
Source: Nutr J. 2020 Jul 2;19:64. doi: 10.1186/s12937-020-00576-2 (PMC7333253; doi:10.1186/s12937-020-00576-2)
Supplement: Supplementary file 1 — Additional file 1: Figure S1. Flow chart of the present investigation Figure S2. Association between serum cobalamin and logarithmically transformed serum folate before and after adjustments for covariables within linear mixed-effects model. [file 12937_2020_576_MOESM1_ESM.pdf]

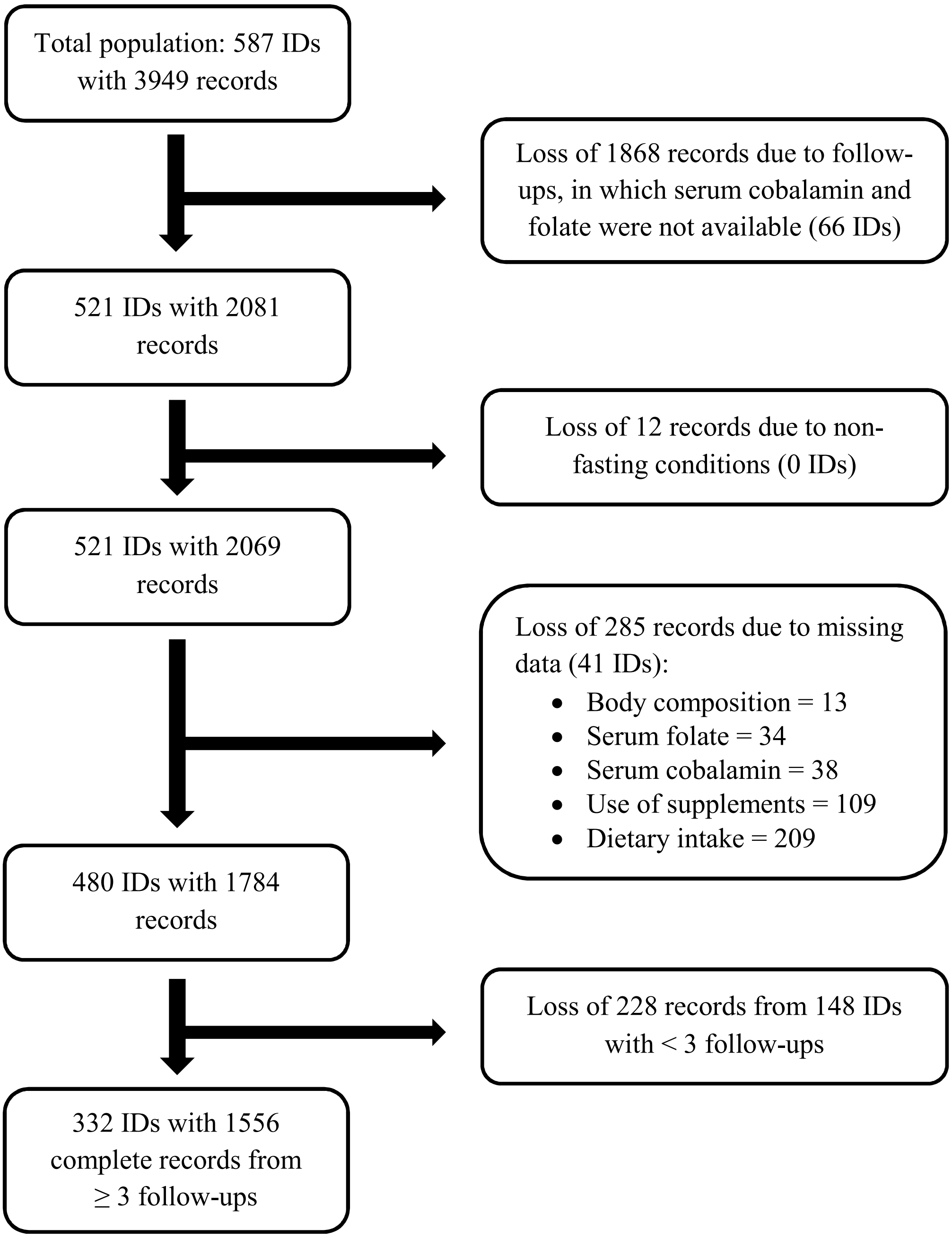

Supplementary Figure 1 Flow chart of the present investigation

The flow chart illustrates the numbers and reasons for excluding subjects (indicated by ID) and/or records. In total, 587 subjects participated in the GISELA study from 1994 to 2014. Duplicate measurements of both serum cobalamin and serum folate were available at follow-ups in 1997, 1998, 2002, 2006, 2010, 2012 and 2014.

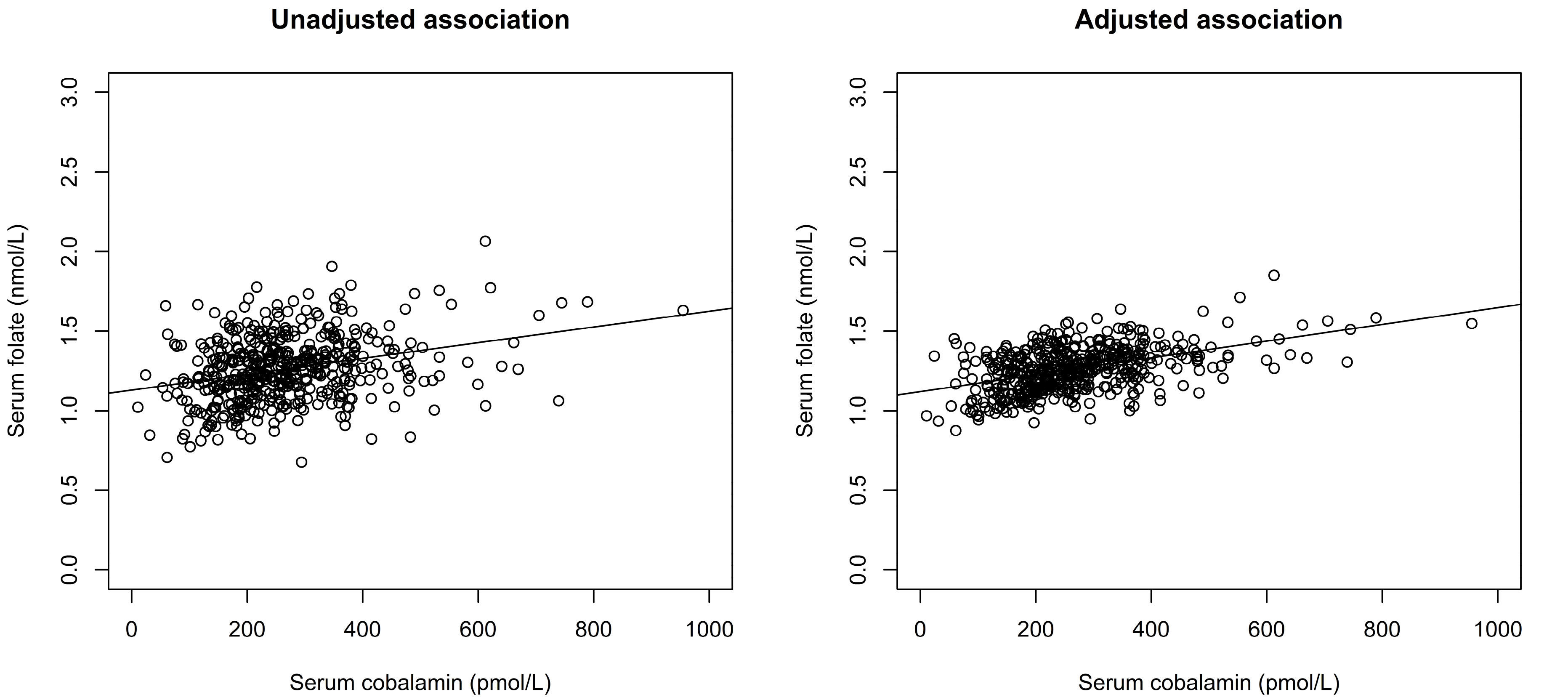

Supplementary Figure 2 Association between serum cobalamin and logarithmically transformed serum folate before and after adjustments for covariables within linear mixed-effects model

On the left side, the unadjusted association between serum cobalamin and logarithmically transformed (log_{10}) serum folate is illustrated. On the right side, the association between the observed serum cobalamin concentrations and the predicted log_{10} serum folate concentrations using the restricted model 4 is illustrated. Both illustrations based on the sample used for applying the restricted model 4. This implies that subjects with lifetime diagnosis of cancer or inflammatory bowel disease and records with serum cobalamin above 1000 pmol/L, use of vitamin B/multi-vitamin supplements or residuals outside of the assumed normal distribution were not investigated. This restriction resulted in a sub sample of 122 subjects, who provided complete data sets on at least three follow-ups.
