# Supplementary material for: Dynamics and interactions of cobalamin and folate status during advanced aging – a longitudinal study in a community-dwelling cohort with multiple follow-ups
Source: Nutr J. 2020 Jul 2;19:64. doi: 10.1186/s12937-020-00576-2 (PMC7333253; doi:10.1186/s12937-020-00576-2)
Supplement: Supplementary file 2 — Additional file 2: Table S1. Predictors of serum cobalamin using linear mixed-effects models with BMI, relative FFM and absolute FM as main effects (n = 332) Table S2. Predictors of serum folate using linear mixed-effects models with BMI, relative FFM and absolute FM as main effects (n = 332) Table S3. Predictors of logarithmically transformed serum cobalamin using linear mixed-effects models without subjects with lifetime diagnosis of cancer/inflammatory bowel disease and records with serum cobalamin > 1000 pmol/L or with outlying residuals Table S4. Predictors of logarithmically transformed serum folate using linear mixed-effects models without subjects with lifetime diagnosis of cancer/inflammatory bowel disease and records with serum cobalamin > 1000 pmol/L or with outlying residuals. [file 12937_2020_576_MOESM2_ESM.pdf]

Supplementary Table 1 Predictors of serum cobalamin using linear mixed-effects models with BMI, relative FFM and absolute FM as main effects (*n* = 332) [a]

	Model with BMI (kg/m²)		Model with FFM (%)		Model with FM (kg)	
	PE [95% CI]	*P* [b]	PE [95% CI]	*P* [b]	PE [95% CI]	*P* [b]
Intercept	325.97 [289.87, 362.06]	<0.001	318.96 [280.10, 357.83]	<0.001	325.09 [288.80, 361.39]	<0.001
Serum folate (nmol/L)	3.54 [2.04, 5.03]	<0.001	3.57 [2.08, 5.07]	<0.001	3.56 [2.06, 5.05]	<0.001
Vitamin B/multi-vitamin supplementation	96.41 [50.23, 142.59]	<0.001	96.64 [50.48, 142.81]	<0.001	96.42 [50.24, 142.60]	<0.001
Age (years)	2.55 [-1.82, 6.91]	0.980	2.67 [-1.68, 7.02]	0.968	2.68 [-1.69, 7.04]	0.970
Cobalamin intake (µg/d)	2.10 [-4.31, 8.52]	1.000	1.97 [-4.43, 8.38]	1.000	2.00 [-4.41, 8.41]	1.000
Folate intake (µg/d)	-0.11 [-0.33, 0.12]	0.998	-0.10 [-0.33, 0.13]	0.999	-0.10 [-0.33, 0.13]	0.998
Alcohol intake (g/d)	-0.78 [-2.80, 1.24]	1.000	-0.72 [-2.73, 1.29]	1.000	-0.74 [-2.76, 1.27]	1.000
Past/current smoking	5.11 [-49.51, 59.72]	1.000	4.59 [-49.93, 59.11]	1.000	4.75 [-49.83, 59.33]	1.000
Male sex	-24.85 [-87.14, 37.43]	1.000	-2.36 [-80.31, 75.58]	1.000	-22.04 [-85.78, 41.70]	1.000
Age x serum folate	0.38 [0.22, 0.54]	<0.001	0.38 [0.22, 0.54]	<0.001	0.38 [0.22, 0.54]	<0.001
Age x supplementation	4.10 [-2.48, 10.69]	0.966	3.98 [-2.59, 10.56]	0.971	4.03 [-2.55, 10.61]	0.971
Age x cobalamin intake	-0.49 [-1.16, 0.18]	0.891	-0.48 [-1.15, 0.18]	0.893	-0.48 [-1.15, 0.18]	0.899
Age x sex	0.13 [-7.21, 7.46]	1.000	-0.03 [-7.36, 7.30]	1.000	0.03 [-7.31, 7.37]	1.000
Sex x serum folate	7.55 [4.92, 10.18]	<0.001	7.54 [4.91, 10.17]	<0.001	7.54 [4.91, 10.17]	<0.001
BMI (kg/m²)	-0.57 [-6.19, 5.06]	1.000				
FFM (%)			-1.91 [-5.90, 2.07]	0.997		
FM (kg)					0.56 [-2.13, 3.25]	1.000
Model fit [c]	0.705		0.704		0.705	
Residual standard deviation	312.13		312.08		312.13	
AIC	22,562		22,561		22,563	

PE Parameter estimate, *95% CI* 95% confidence interval, *BMI* body mass index, *FFM* fat-free mass, *FM* fat mass; *AIC* Akaike's information criterion

[a] Linear mixed-effects models including serum cobalamin concentrations as dependent variable, random effects of age and subject and centered metric independent variables. The model based on the initial model 2, which considered as fixed effects: serum folate, vitamin B/multi-vitamin supplementation, age, cobalamin intake, folate intake, alcohol intake, smoking, sex and effect modifications by sex and age. For this sub analysis, the initially included absolute FFM was replaced by BMI, relative FFM and absolute FM, respectively.

[b] Denotes *P* values after adjusting for simultaneous inference

[c] Correlation between observed and predicted serum cobalamin concentrations

Supplementary Table 2 Predictors of serum folate using linear mixed-effects models with BMI, relative FFM and absolute FM as main effects (*n* = 332) [a]

	Model with BMI (kg/m²)		Model with FFM (%)		Model with FM (kg)	
	PE [95% CI]	*P* [b]	PE [95% CI]	*P* [b]	PE [95% CI]	*P* [b]
Intercept	22.88 [21.54, 24.22]	<0.001	23.40 [21.95, 24.85]	<0.001	22.99 [21.64, 24.34]	<0.001
Serum cobalamin (pmol/L)	0.006 [0.003, 0.008]	<0.001	0.006 [0.003, 0.008]	<0.001	0.006 [0.003, 0.008]	<0.001
Vitamin B/multi-vitamin supplementation	6.49 [4.77, 8.21]	<0.001	6.47 [4.75, 8.18]	<0.001	6.49 [4.77, 8.21]	<0.001
Age (years)	0.21 [0.04, 0.38]	0.221	0.22 [0.05, 0.39]	0.171	0.21 [0.04, 0.38]	0.222
Cobalamin intake (µg/d)	-0.16 [-0.40, 0.08]	0.952	-0.16 [-0.40, 0.08]	0.933	-0.16 [-0.40, 0.08]	0.942
Folate intake (µg/d)	0.02 [0.01, 0.03]	0.001	0.02 [0.01, 0.03]	0.001	0.02 [0.01, 0.03]	0.001
Alcohol intake (g/d)	<0.01 [-0.08, 0.08]	1.000	0.01 [-0.07, 0.09]	1.000	0.01 [-0.07, 0.09]	1.000
Past/current smoking	-1.16 [-3.27, 0.95]	0.989	-1.09 [-3.21, 1.02]	0.993	-1.08 [-3.19, 1.04]	0.994
Male sex	-0.98 [-3.28, 1.33]	0.999	-2.73 [-5.68, 0.21]	0.614	-1.45 [-3.82, 0.92]	0.971
Age x serum cobalamin	<0.01 [>-0.01, <0.01]	0.934	<0.01 [>-0.01, <0.01]	0.930	<0.01 [>-0.01, <0.01]	0.932
Age x supplementation	0.26 [0.01, 0.50]	0.447	0.25 [<0.01, 0.50]	0.476	0.25 [<0.01, 0.50]	0.479
Age x folate intake	0.01 [>-0.01, <0.01]	0.966	<0.01 [>-0.01, <0.01]	0.970	<0.01 [>-0.01, <0.01]	0.970
Age x sex	-0.10 [-0.39, 0.19]	1.000	-0.10 [-0.39, 0.19]	1.000	-0.10 [-0.39, 0.19]	1.000
Sex x serum cobalamin	0.01 [0.01, 0.02]	<0.001	0.01 [0.01, 0.02]	<0.001	0.01 [0.01, 0.02]	<0.001
BMI (kg/m²)	-0.23 [-0.45, -0.01]	0.441				
FFM (%)			0.15 [-0.01, 0.30]	0.572		
FM (kg)					-0.09 [-0.20, 0.02]	0.757
Model fit [c]	0.613		0.617		0.617	
Residual standard deviation	13.34		13.31		13.32	
AIC	12,531		12,533		12,534	

PE Parameter estimate, *95% CI* 95% confidence interval, *BMI* body mass index, *FFM* fat-free mass, *FM* fat mass, *AIC* Akaike's information criterion

[a] Linear mixed-effects models including serum folate concentrations as dependent variable, random effects of age and subject and centered metric independent variables. The model based on the initial model 2, which considered as fixed effects: serum cobalamin, vitamin B/multi-vitamin supplementation, age, folate intake, cobalamin intake, alcohol intake, smoking, sex and effect modifications by sex and age. For this sub analysis, the initially included absolute FFM was replaced by BMI, relative FFM and absolute FM, respectively.

[b] Denotes *P* values after adjusting for simultaneous inference

[c] Correlation between observed and predicted serum folate concentrations

Supplementary Table 3 Predictors of logarithmically transformed serum cobalamin using linear mixed-effects models without subjects with lifetime diagnosis of cancer/inflammatory bowel disease and records with serum cobalamin > 1000 pmol/L or with outlying residuals [a]

	Model 2 with total sample (*n* = 237)		Model 3 with serum creatinine (*n* = 144)		Model 4 without users of vitamin B/multi-vitamin supplements (*n* = 121)	
	PE [95% CI]	*P* [b]	PE [95% CI]	*P* [b]	PE [95% CI]	*P* [b]
Intercept	2.4E+00 [2.4E+00, 2.5E+00]	<0.001	2.4E+00 [2.4E+00, 2.4E+00]	<0.001	2.4E+00 [2.3E+00, 2.4E+00]	<0.001
Serum folate (nmol/L)	4.4E-03 [3.6E-03, 5.2E-03]	<0.001	3.8E-03 [2.8E-03, 4.8E-03]	<0.001	4.3E-03 [3.0E-03, 5.6E-03]	<0.001
Vitamin B/multi-vitamin supplementation	2.4E-02 [5.8E-04, 4.8E-02]	0.459	3.3E-02 [-2.1E-03, 6.8E-02]	0.614		
Age (years)	-6.5E-04 [-2.7E-03, 1.4E-03]	1.000	2.1E-03 [-1.4E-03, 5.7E-03]	0.979	-1.4E-04 [-4.0E-03, 3.7E-03]	1.000
Fat-free mass (kg)	-2.5E-03 [-6.6E-03, 1.5E-03]	0.962	-3.7E-03 [-8.8E-03, 1.4E-03]	0.905	-3.6E-03 [-9.0E-03, 1.9E-03]	0.930
Cobalamin intake (µg/d)	4.5E-03 [1.1E-03, 8.0E-03]	0.132	3.6E-03 [-6.1E-04, 7.8E-03]	0.747	1.8E-03 [-2.8E-03, 6.4E-03]	0.999
Folate intake (µg/d)	-4.9E-05 [-1.7E-04, 7.1E-05]	0.999	-6.7E-05 [-2.3E-04, 9.9E-05]	1.000	-1.8E-05 [-2.1E-04, 1.8E-04]	1.000
Alcohol intake (g/d)	-9.6E-05 [-1.4E-03, 1.2E-03]	1.000	1.6E-04 [-1.7E-03, 2.0E-03]	1.000	3.8E-05 [-1.8E-03, 1.9E-03]	1.000
Past/current smoking	1.6E-03 [-4.5E-02, 4.8E-02]	1.000	2.6E-03 [-5.5E-02, 6.0E-02]	1.000	-1.0E-02 [-7.5E-02, 5.4E-02]	1.000
Male sex	-1.4E-02 [-9.7E-02, 6.9E-02]	1.000	7.9E-03 [-1.0E-01, 1.2E-01]	1.000	2.8E-02 [-8.9E-02, 1.5E-01]	1.000
Age x serum folate	-1.2E-04 [-2.2E-04, -1.4E-05]	0.300	-9.8E-05 [-2.3E-04, 3.6E-05]	0.901	-1.3E-04 [-2.9E-04, 3.2E-05]	0.776
Age x supplementation	2.5E-03 [-8.7E-04, 5.8E-03]	0.877	-1.8E-04 [-5.5E-03, 5.2E-03]	1.000		
Age x cobalamin intake	-3.1E-04 [-6.3E-04, 7.2E-06]	0.534	-2.4E-04 [-6.9E-04, 2.0E-04]	0.990	-6.2E-04 [-1.1E-03, -1.0E-04]	0.206
Age x sex	-2.3E-03 [-5.8E-03, 1.2E-03]	0.947	-5.4E-03 [-1.2E-02, 8.4E-04]	0.732	-1.3E-03 [-8.2E-03, 5.6E-03]	1.000
Sex x serum folate	2.6E-03 [1.0E-03, 4.2E-03]	0.016	9.7E-04 [-6.5E-04, 2.6E-03]	0.979	5.4E-04 [-1.4E-03, 2.5E-03]	1.000
Serum creatinine (mg/dL)			4.0E-02 [-3.5E-02, 1.2E-01]	0.992	2.8E-02 [-5.3E-02, 1.1E-01]	1.000
Model fit [c]	0.886		0.915		0.916	
Residual standard deviation	0.114		0.104		0.101	
AIC	-904		-428		-348	

PE Parameter estimate, *95% CI* 95% confidence interval, *AIC* Akaike's information criterion

[a] Data represent the results of the linear mixed-effects models including logarithmically transformed (log_{10}) serum cobalamin concentrations as dependent variable, random effects of age and subject and centered metric independent variables. Model 2 considered as fixed effects: serum folate, vitamin B/multi-vitamin supplementation, age, absolute fat-free mass, cobalamin intake, folate intake, alcohol intake, smoking, sex and effect modifications by sex and age. Model 3 based on model 2 and considered serum creatinine as additional fixed effect. Model 4 based on model 3 but excluded records, in which use of vitamin B/multi-vitamin supplements was reported.

[b] Denotes *P* values after adjusting for simultaneous inference

[c] Correlation between observed and predicted log_{10} serum cobalamin concentrations

Supplementary Table 4 Predictors of logarithmically transformed serum folate using linear mixed-effects models without subjects with lifetime diagnosis of cancer/inflammatory bowel disease and records with serum cobalamin > 1000 pmol/L or with outlying residuals [a]

	Model 2 with total sample (*n* = 235)		Model 3 with serum creatinine (*n* = 145)		Model 4 without users of vitamin B/multi-vitamin supplements (*n* = 122)	
	PE [95% CI]	*P* [b]	PE [95% CI]	*P* [b]	PE [95% CI]	*P* [b]
Intercept	1.3E+00 [1.2E+00, 1.3E+00]	<0.001	1.3E+00 [1.2E+00, 1.3E+00]	<0.001	1.3E+00 [1.2E+00, 1.3E+00]	<0.001
Serum cobalamin (pmol/L)	5.3E-04 [4.3E-04, 6.3E-04]	<0.001	6.1E-04 [4.7E-04, 7.6E-04]	<0.001	6.4E-04 [4.7E-04, 8.2E-04]	<0.001
Vitamin B/multi-vitamin supplementation	8.3E-02 [5.5E-02, 1.1E-01]	<0.001	1.1E-01 [6.4E-02, 1.5E-01]	<0.001		
Age (years)	1.2E-03 [-1.2E-03, 3.5E-03]	0.995	6.8E-04 [-3.0E-03, 4.4E-03]	1.000	1.7E-03 [-2.4E-03, 5.8E-03]	0.998
Fat-free mass (kg)	-1.3E-03 [-5.3E-03, 2.7E-03]	1.000	-3.4E-03 [-8.8E-03, 2.0E-03]	0.967	-2.2E-03 [-8.1E-03, 3.6E-03]	0.999
Cobalamin intake (µg/d)	-5.4E-03 [-9.0E-03, -1.7E-03]	0.053	-5.3E-03 [-9.9E-03, -8.1E-04]	0.264	-5.6E-03 [-1.1E-02, -6.6E-04]	0.285
Folate intake (µg/d)	4.0E-04 [2.6E-04, 5.5E-04]	<0.001	4.4E-04 [2.3E-04, 6.5E-04]	<0.001	5.2E-04 [2.8E-04, 7.6E-04]	<0.001
Alcohol intake (g/d)	-2.9E-04 [-1.7E-03, 1.1E-03]	1.000	1.3E-04 [-1.9E-03, 2.1E-03]	1.000	7.2E-04 [-1.4E-03, 2.8E-03]	1.000
Past/current smoking	1.4E-03 [-4.1E-02, 4.3E-02]	1.000	-1.3E-02 [-7.0E-02, 4.3E-02]	1.000	2.4E-02 [-4.1E-02, 8.8E-02]	1.000
Male sex	5.9E-03 [-7.4E-02, 8.5E-02]	1.000	-5.5E-03 [-1.2E-01, 1.1E-01]	1.000	-4.7E-02 [-1.7E-01, 7.5E-02]	0.999
Age x serum cobalamin	6.1E-06 [-5.1E-06, 1.7E-05]	0.988	-2.1E-05 [-3.8E-05, -3.5E-06]	0.239	-3.6E-05 [-5.7E-05, -1.5E-05]	0.011
Age x supplementation	1.9E-03 [-2.0E-03, 5.8E-03]	0.996	7.6E-04 [-5.4E-03, 6.9E-03]	1.000		
Age x folate intake	1.6E-05 [1.7E-07, 3.1E-05]	0.481	1.8E-05 [-6.5E-06, 4.3E-05]	0.894	2.6E-05 [-3.1E-06, 5.5E-05]	0.635
Age x sex	-7.2E-04 [-4.8E-03, 3.3E-03]	1.000	2.3E-03 [-4.0E-03, 8.6E-03]	1.000	-3.4E-03 [-1.1E-02, 4.0E-03]	0.996
Sex x serum cobalamin	2.8E-04 [7.1E-05, 4.9E-04]	0.115	3.9E-04 [1.0E-04, 6.8E-04]	0.106	4.0E-04 [5.5E-05, 7.5E-04]	0.253
Serum creatinine (mg/dL)			2.3E-01 [1.4E-01, 3.2E-01]	<0.001	2.5E-01 [1.6E-01, 3.4E-01]	<0.001
Model fit [c]	0.792		0.827		0.834	
Residual standard deviation	0.150		0.147		0.140	
AIC	-586		-220		-183	

PE Parameter estimate, *95% CI* 95% confidence interval, *AIC* Akaike's information criterion

[a] Data represent the results of the linear mixed-effects models including logarithmically transformed (log_{10}) serum folate concentrations as dependent variable, random effects of age and subject and centered metric independent variables. Model 2 considered as fixed effects: serum cobalamin, vitamin B/multi-vitamin supplementation, age, absolute fat-free mass, cobalamin intake, folate intake, alcohol intake, smoking, sex and effect modifications by sex and age. Model 3 based on model 2 and considered serum creatinine as additional fixed effect. Model 4 based on model 3 but excluded records, in which use of vitamin B/multi-vitamin supplements was reported.

[b] Denotes *P* values after adjusting for simultaneous inference

[c] Correlation between observed and predicted log_{10} serum folate concentrations
